# Spectrum of Germline Pathogenic Variants in BRCA1/2 Genes in the Apulian Southern Italy Population: Geographic Distribution and Evidence for Targeted Genetic Testing

**DOI:** 10.3390/cancers13184714

**Published:** 2021-09-21

**Authors:** Margherita Patruno, Simona De Summa, Nicoletta Resta, Mariapia Caputo, Silvia Costanzo, Maria Digennaro, Brunella Pilato, Rosanna Bagnulo, Antonino Pantaleo, Cristiano Simone, Maria Iole Natalicchio, Elisabetta De Matteis, Paolo Tarantino, Stefania Tommasi, Angelo Paradiso

**Affiliations:** 1 Center for Hereditary Tumors Research, Istituto Tumori Bari, Giovani Paolo II, IRCCS, 70124 Bari, Italy; s.costanzo@oncologico.bari.it (S.C.); m.digennaro@oncologico.bari.it (M.D.); a.paradiso@oncologico.bari.it (A.P.); 2 Molecular and Pharmacogenetics Diagnostic Laboratory, IRCCS-IstitutoTumori “Giovanni Paolo II”, 70124 Bari, Italy; m.caputo@oncologico.bari.it (M.C.); b.pilato@oncologico.bari.it (B.P.); s.tommasi@oncologico.bari.it (S.T.); 3 Medical Genetics Unit, Department of Biomedical Sciences and Human Oncology, “Aldo Moro” University of Bari, Policlinico Hospital Bari, 70124 Bari, Italy; nicoletta.resta@uniba.it (N.R.); rosanna.bagnulo@uniba.it (R.B.); antonino.pantaleo@uniba.it (A.P.); cristiano.simone@uniba.it (C.S.); 4 Medical Genetics, National Institute of Gastroenterology “S. de Bellis” Research Hospital, Castellana Grotte, 70013 Bari, Italy; 5 Section of Clinic Pathology, OO.RR., 71122 Foggia, Italy; iole.nat@tiscali.it; 6 Oncology Unit, “Vito Fazzi” Hospital, 73100 Lecce, Italy; dr.dematteis.elisabetta@gmail.com; 7 Medical Genetics Unit, “Vito Fazzi” Hospital, 73100 Lecce, Italy; bt.tarantino@gmail.com

**Keywords:** BRCA1, BRCA2, hereditary breast and ovarian cancer, Apulian population, geographic distribution, southern Italy

## Abstract

**Simple Summary:**

*BRCA1* and *BRCA2* are two major high-penetrance breast/ovarian cancer predisposition genes, whose mutations can lead to high risk and early onset of breast and ovarian cancer. Numerous studies are focused on spectrum and prevalence of *BRCA1/2* mutations worldwide. This is the first study that exclusively focused on native Apulian probands. We found that ten recurrent *BRCA1/2* pathogenic variants account for more than half of the patients with proven HBOC syndrome from Apulia. Besides BRCA1 c.5266dupC, which is present in significant numbers in every Apulian province, the other PVs occur at a high frequency in some areas and not others. In-depth knowledge of the mutation spectrum of the target population and of the relatively small number of recurrent mutations is crucial to develop a specific cost-effective strategy for mutation screening and a program for breast–ovarian cancer control and prevention through more liberal, yet rational, genetic testing and counseling.

**Abstract:**

BRCA1/2-associated hereditary breast and ovarian cancer is the most common form of hereditary breast and ovarian cancer and occurs in all ethnicities and racial populations. Different BRCA1/BRCA2 pathogenic variants (PVs) have been reported with a wide variety among populations. In this study, we retrospectively analyzed prevalence and geographic distribution of pathogenic germline BRCA1/2 variants in families from Apulia in southern Italy and evaluated the genotype–phenotype correlations. Data were collected from Oncogenetic Services present in Apulian hospitals and a shared database was built containing Apulian native probands (*n* = 2026) that had undergone genetic testing from 2004 to 2019. PVs were detected in 499 of 2026 (24.6%) probands and 68.5% of them (342 of 499) were in the BRCA1 gene. We found 65 different PVs in BRCA1 and 46 in BRCA2. There were 10 most recurrent PVs and their geographical distribution appears to be significantly specific for each province. We have assumed that these PVs are related to the historical and geopolitical changes that occurred in Apulia over time and/or to a “founder effect”. Broader knowledge of BRCA1/2 prevalence and recurring PVs in specific geographic areas could help establish more flexible genetic testing strategies that may enhance our ability to detect high-risk subjects.

## 1. Introduction

Hereditary Breast and Ovarian Cancer Syndrome (HBOC) is an autosomal dominantly inherited condition caused by the presence of germline pathogenic variants (PVs) in the BRCA1 and BRCA2 genes. The predominance of BRCA1/2 pathogenic variants in the general population (excluding the Ashkenazim) is estimated to be 1:400 to 1:500 [1]. HBOC is characterized by an increased risk of developing breast and/or ovarian cancer. In fact, it manifests with the onset of multiple cases of breast or ovarian cancers in the same parental line, usually at an early age and/or with bilateral localization.

The lifetime risk of developing one of these tumors varies with the mutated gene. In fact, in the general population’s lifetime, the risk of developing breast and ovarian cancer is 12 and 1.5%, respectively [2], while for a woman carrying a PV of the BRCA1 gene, the risk grows to 67 and 40%, respectively [3]. Moreover, BRCA1 carriers have a 27% risk of developing contralateral breast cancer within 5 years from the initial diagnosis [3]. For a woman carrying a PV of the BRCA2 gene, the lifetime risk of developing a breast and ovarian cancer is about 66 and 12%, respectively, and the risk of developing contralateral breast cancer is about 12% in the 5 years from the initial diagnosis [3].

HBOC syndrome tumors seem to be associated with a typical histological and molecular set of features. Undifferentiated (G3) basal-like ductal breast cancers (triple-negative) are the most recurrent in BRCA1 carriers [4], while fewer data are available about BRCA2 carriers, although BRCA2 tumors seem to be more similar to sporadic breast cancer both for histological and molecular characteristics [5]. In ovarian cancer, the prevalence of BRCA1/2 PVs is high and increases up to 23–25% in the high-grade serous histotype [6,7].

In males, BRCA1 and BRCA2 PVs are responsible for the increased risk (higher in BRCA2 carriers) of developing breast and prostatic cancer [8].

There is also some evidence that both males and females have an increased risk of developing pancreatic cancer with a lifetime risk of 2–7% and 1–3% for BRCA2 and BRCA1 carriers, respectively [8].

Given their significantly increased risk of cancer compared to the general population, it is important that individuals carrying BRCA1/2 PVs be identified to provide them with access to specific clinical and diagnostic surveillance programs. Magnetic resonance imaging screening from an early age and risk reducing measures such as chemoprevention and prophylactic surgery have been shown to substantially decrease HBOC-associated morbidity and mortality [9,10]. Moreover, the identification of BRCA 1/2 PVs carriers may lead oncologists to prefer tailored pharmacotherapies for BRCA1/2 associated malignancies, since the diagnostic assessment for hereditary cancer syndromes also plays a crucial role in oncological care [11].

BRCA1/2 analysis is performed in all suspected cases of HBOC, in accordance with the NCCN guidelines [12].

Since the BRCA genes are large genes, hundreds of mutations have been identified in them. Most pathogenic variants (≥80%) are detected through whole-gene sequencing, with an additional 10% detected through deletion/duplication analysis [13]. Despite the high variability of family PVs, some PVs have frequently been observed in specific geographic locations and even in ethnicities [14].

Full BRCA1 and BRCA2 gene screening still remains a huge labor- and time-consuming challenge due to the large size of the genes. Furthermore, different PVs or variants of unknown significance (VUS) and the complexity of large genomic rearrangements (LGRs) require an especially technical approach, so this procedure remains too complex and expensive to cover a broader target (e.g., all breast or ovarian cancer patients). 

Acknowledgement of the prevalence of BRCA1/2 and of recurrent PVs in specific geographic areas could be instrumental in developing a new, more cost-effective population-based genetic approach [15,16].

The aim of the present exploratory study is to describe the prevalence and distribution of BRCA1/2 PVs in Apulian population in order to evaluate the possibility to perform targeted mutational BRCA1/2 screening in the future.

## 2. Materials and Methods

### 2.1. Data Set

We retrospectively collected and analyzed clinical and genetic information of 2228 HBOC patients who received Oncogenetic Counseling (OGC) in Apulia from 2004 to 2019 and had been tested for germline PVs in BRCA1/2 genes following NCCN testing criteria [12]. Due to the periodic updating of these guidelines, we point out that the genetic testing criteria have changed over time based on these updates.

All the patients were recruited within the setting of oncogenetic counselling at the Genetic Services present in the following Apulian Hospitals: IRCCS IstitutoTumori “Giovanni Paolo II” in Bari, the Policlinico University Hospital in Bari, the “OspedaliRiuniti” University Hospitalin Foggia, and the “Vito Fazzi” Hospital in Lecce. Written informed consent for genetic testing and research was obtained from allthe participants. The following information was collected for each patient: gender, age (calculated from their birthdate to the end of data collection), date and place of birth, place of residence, health status (cancer diagnosis), number of cancers, cancer site, age at onset, cancer histotype, and BRCA1/2 genotype. Since our aim was to analyze the prevalence and distribution of BRCA1/2 PVs across the Apulian provinces, 202/2228 patients were excluded from our sample because they were not natives of Apulia. Thus, a total of 2026 patients were included in our study.

Based on place of birth and place of residence, the index cases were grouped within the 6 Apulian provinces. 

The cancer sites were grouped into the following 6 categories: breast, ovary, prostate, pancreas, melanoma, other. 

Breast cancer histotypes were divided into 5 subgroups: ductal carcinoma in situ, lobular carcinoma in situ, infiltrating ductal carcinoma, infiltrating lobular carcinoma, other breast cancer histotypes (e.g., tubular, papillary, medullary, mucinous, sarcomatoid carcinoma, and mammary Paget disease). 

Ovarian cancer histotypes were divided into 3 subgroups: high-grade serous carcinoma, endometrioid, other histotype (e.g., mucinous, low-grade serous and clear-cell carcinoma). 

No histological sub-classification was used for the other cancer sites.

### 2.2. Molecular BRCA1/2 Analysis

A peripheral blood sample was taken from each patient for BRCA1/2 genotyping. Mutational analysis of coding sequences and intron-exon boundaries of BRCA1/2 genes was carried out with denaturing high-performance liquid chromatography (DHPLC) or next generation sequencing (NGS), depending on which technique was present at the time. The presence of large genomic rearrangements (LGRs) was detected with Multiplex Ligation-dependent Probe Amplification (MLPA). 

Since the cases occurred over the period 2004–2019, all the detected BRCA1/2 genetic variants were rechecked following the Evidence-based Network for the Interpretation of Germline Mutant Alleles (ENIGMA) Consortium guidelines [17] and classified according to the International Agency for Research on Cancer (IARC) recommendation [18] using a system that divides the variants into 5 classes: benign (class I), likely benign (class II), variant of uncertain significance (VUS, class III), likely pathogenic (class IV), pathogenic (class V). All the mutations identified were named in accordance with the Human Genome Variation Society (HGVS) nomenclature [19]. 

On the basis of the BRCA1/2 genotype, our sample was divided into 3 classes: carrier (when pathogenic/likely-PVs of BRCA1/2 were detected), non-carrier (when no mutation was identified), and VUS (when a variant of uncertain significance was identified). 

### 2.3. Statistical Analysis

Proportions of PVs were compared using the proportion Z test with R v.3.6.2. Lolliplots displaying the location of PVs along protein domains were depicted with the R packageGenVisR [20].

## 3. Results

### 3.1. Data Set Description

As reported in Table 1, the total cohort of 2026 probands consists of 1980 cancer patients and 46 (2%) healthy individuals at-risk; 1947 female and 79 (4%) male. 

Their mean age was 55 years (range 21–92 years). 

Genetic BRCA1/2 testing identified a pathogenic variant in 499 of the 2026 (24.5%) probands, a variant of uncertain significance (VUS) was found in 131 (6.5%), and no mutation was found in 1396 (69%). 

Among the 499 carriers, 342 (68.4%) had PVs in the BRCA1 gene and 158 (31.6%) in the BRCA2 gene. One proband presented both BRCA1 and BRCA2 PVs.

Forty-eight (36.5%) of the 131 VUS were in BRCA1 and 83 (63.5%) were in BRCA2.

Table 2 breaks down the numbers of probands by the Apulian province where they were born and reports their BRCA1/2 status. The number of native probands of the Foggia province was not very representative of that area due to issues with data collection.

### 3.2. Phenotype Description

Clinical data were available for 1950 of 1980 affected probands. A total of 297 of the 1950 affected patients (15.23%) had been diagnosed with 2 cancers; 36 (1.84%) with 3 different cancers. Only 1 patient developed 4 primitive cancers. The distribution of the types of tumors by mutational status is displayed in Table 3. In particular, the VUS-carriers predominantly had breast cancer (84.9%). This proportion is significantly higher (*p*-value < 0.0001) when compared to the percentages of carriers (67.4%) and non-carriers (82.3%) (Table 3).

In detail, we observed that ductal breast cancer was significantly more frequent (*p*-value < 0.0001) in carriers (84.3%) than in non-carriers and VUS-carriers (74.28% and 75.26%, respectively). Lobular breast cancer was significantly more frequent (*p*-value = 0.004) in non-carriers (7.67%) than in carriers (2.54%) and VUS-carriers (5.37%) (Table 4). Moreover, the percentage of carriers with ovarian cancer (22.1%) was significantly higher than that of non-carriers and VUS-carriers (*p*-value < 0.0001) (Table 3). Regarding the histopathological subtypes, a statistical trend (*p*-value = 0.08) indicated that the number of probands affected by serous-papillary ovarian cancer was significantly higher amongst carriers (81.9%) than non-carriers (77.91%) and VUS-carriers (58.82%) (Table 5).

### 3.3. Genotype Description

Mutational analysis revealed that 97 different BRCA1/2 VUS, 32 of which were detected in the BRCA1 gene and 65 in the BRCA2 gene, were present in 2026 unrelated tested probands [Table S1]. Moreover, 100 different BRCA1/2 PVs –56 in BRCA1 and 44 in BRCA2– and 11 different pathogenic LGRs, 9 of which were in BRCA1 and 2 in BRCA2, were detected in the same cohort. 

**Figure 1:**
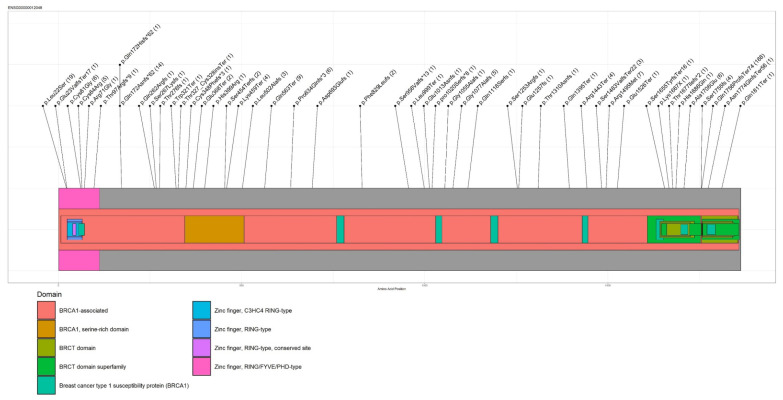
Lolliplot representing BRCA1 PVs. Numbers in brackets indicate the number of occurrence of PVs.

**Figure 2:**
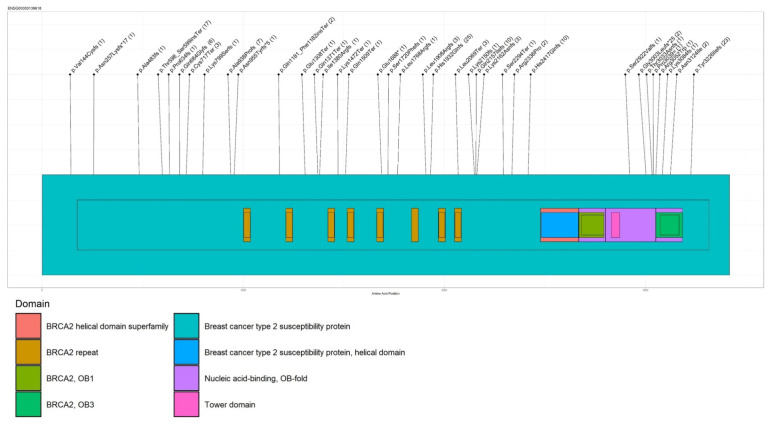
Lolliplot representing BRCA2 PVs. Numbers in brackets indicate the number of occurrence of PVs.

The different PVs identified in BRCA1 and BRCA2 are shown in Figure 1 and Figure 2, respectively. Furthermore, BRCA 1/2 large genomic rearrangements and BRCA 1/2 splicing variants are shown in Table S2. 

The most recurrent PV identified in BRCA1 carriers was c.5266dupC (p.Gln1756ProfsTer74) that was detected in 188/342 (54.9%) carriers. It was also the most frequently detected PV in all carriers (188/499, 37.6%).

Other recurrent PVs identified in the whole cohort are shown in Table 6.

These PVs were responsible for increased genetic risk in 324 unrelated probands, representing 65% (324/499) of the total number of cases with identified BRCA1/2 PVs in Apulia.

Table 7 shows the frequencies of the aforementioned PVs from the ExAC database, both for Europe and the full dataset, evidencing their enrichment among Apulian carriers. Their proportions were also statistically compared among Apulian provinces. 

We observed that BRCA1: c.5266dupC, BRCA1:c.65T > C, BRCA1:c.514delC and BRCA1:c.4484G > T are significantly enriched in the BAT province (grouping the towns of Barletta, Andria, and Trani) (*p*-value < 0.0001), Bari (*p*-value = 0.018), Lecce (*p*-value < 0.0001), and Taranto (*p*-value < 0.0001) (Table 7).

The BRCA2 PVs: c.5796_5797delTA, c.9676delT, c.1796_1800delCTTAT, and c.8755-1G > A are significantly frequent in BAT (*p*-value < 0.0001), Lecce (*p*-value < 0.0001), Bari (*p*-value = 0.025), and Lecce (*p*-value = 0.0009) (Table 7).

In anattempt to better localize the enriched mutations, we compared the distribution of the mutations in the cities of the province where they were found to be significant. Regarding BRCA1 PVs, c.5266dupC, enriched in theBAT province, is more frequent in Andria (20/46, 43.46%); c.65T > C is exclusively present in Bari (12/12, 100%); c.514delC is enriched in Nardò (3/14; 21.42%) and c.4484G > T is more frequent in Laterza (3/5; 60%). BRCA2 PVs were also explored in the cities of the province where they were significant. c.5796_5797delTA is more frequent in Bisceglie (4/10; 40%); c.9676delT is enriched in Lecce (3/17; 17.64%); c.1796_1800delCTTAT is more frequent in Bitetto (3/12; 25%) and c.8755-1G > A is enriched in Casarano (3/10; 30%).

## 4. Discussion

In this study, we retrospectively collected and analyzed the clinical information of 2026 HBOC Apulian patients enrolled at Genetic Services present in several Apulian Hospitals: IRCCS IstitutoTumori “Giovanni Paolo II” in Bari, the Policlinico University Hospital in Bari, the “OspedaliRiuniti” University Hospitalin Foggia, and the “Vito Fazzi” Hospital in Lecce. To our knowledge, this is the first study that exclusively focused on native Apulian probands with a suspected HBOC syndrome according to the NCCN guidelines. The strengths of the study include the large sample size and its homogeneity. Other similar Italian studies had been published previously but with a less homogeneous sample than ours, since they enrolled both probands and relatives [21,22] as well as patients from different Italian regions [23,24,25]. Therefore, our work depicts the actual prevalence, spectrum, and distribution of BRCA1/2 variants in the Apulia region. 

A pathogenic variant was identified in 24.5% probands. This figure is higher than the prevalence reported in other Italian regions: 10% in Sardinia [26], 14.8% in Sicily [21], 13.8% in Central Italy [22], and 18.9% in northeastern Italy [23]. While interesting, this finding is severely limited by the different genetic test access criteria used in the previous reports. The percentage of BRCA1 PVs found in our study was higher than that of the BRCA2 PVs (68.4% and 31.6, respectively). This distribution differs from that of other Italian regions [21,22,23] where the percentage of PVs observed in the 2 genes is generally the same, except for Sardinia where BRCA2 PVs are more frequent [26]. 

In the Apulian patients who underwent genetic testing, we observed 100 distinct PVs (56 in BRCA1 and 44 in BRCA2). Most of them were identified in 1 to 3 probands, while only 10 PVs (5 in BRCA1 and 5 in BRCA2) were frequently recorded in non-related probands and globally recorded in 67% of BRCA1/2 carriers. Comparing the prevalence of these 10 pathogenic variants in Apulia, across Europe and worldwide [27,28], a high recurrence is observed even though our sample size was smaller. Further research on a larger sample is required to confirm or refute this observation.

BRCA1 c.5266dupC is one of the most frequently reported PV worldwide [27]. This mutation was originally described as a founder mutation in the Ashkenazi Jewish population; however, it is also present in many European countries with a low proportion of individuals who self-identify as Jewish. According to Hamel et al. [29], BRCA1 c.5266dupC was a common European mutation before it became an Ashkenazi Jewish founder mutation. It appears to have originated 1800 years ago in Scandinavia or in what is now northern Russia and then spread to various populations; it seems have entered the Ashkenazi Jewish population in Poland approximately 400–500 years ago. 

In our sample, BRCA1 c.5266dupC was present in 54.9% of Apulian BRCA1 carriers and in 37.6% of Apulian BRCA1/2 carriers. These percentages are higher than the rates previously reported in Italy.Hamel et al. [29] described the presence of BRCA1 c.5266dupC in 7.5% of Italian BRCA1 carriers, while Ramunas [14] reported the same variant in 3% of Italian BRCA1/2 carriers. Other Italian studies described the BRCA1/2 mutation spectrum in specific geographical areas as well as in their samples and reported that BRCA1 c.5266dupC was not native to those locations and had been imported from southern Italy [23,24]. Figlioli et al. [24] believe this variant originated in Apulia and spread to Lombardy in northern Italy due to carriers’ mobility from Apulia to that northern region in search of health care. 

Once the prevalence of BRCA1 c.5266dupC in Apulia is compared with the data in the literature, it can be argued that the high presence of this mutation in Apulia is due to the geopolitical history of the region. Colonization of the region by the Normans around 1000 D.C. and the presence of numerous Jewish communities contributed to the spread of BRCA1 c.5266dupC in the area. In addition, the prevalence of BRCA1 c.5266dupC in the different Apulian provinces was significantly higher in the BAT province, comprising the three towns of Barletta, Andria, and Trani, with the latter being known as “the capital of Judaism” in Apulia [30].

Similarly to the rest of Europe [27], BRCA1 c.1687C > T represents one of the five most common mutations in the BRCA1 gene also in Apulia.No significant difference was observed in the distribution of this PV in the 6 Apulian provinces. To our knowledge, this mutation was reported in Italy only by Santonocito et al. [25] who described BRCA1/2 germline PVs in patients from central and southern Italy. 

In our sample, BRCA1 c.514delC was observed exclusively in the Lecce province. To our knowledge this variant has been described elsewhere by Palmero et al. [31] only in one Brazilian proband. In Italian studies, high levels of recurrence have been reported for this mutation in Sicily, specifically in the geographical areas that include the cities of Palermo and Messina [21]. Other Italian groups [24,25] have reported this variant but have described it as beingimported from Apulia and Sicily subsequent topatients moving to Rome or Milan to seek healthcare. Further studies are needed to understand the actual origin of this variant.

BRCA1 c.65T > C was first observed in a Japanese woman of Jewish origin [32]. There is no data regarding its prevalence in the population database and no other Italian group has reported this variant except us. In our sample, BRCA1 c.65T > C was one of the most frequent PVs and showed a significant enrichment in the city of Bari. All these considerations point to the likelihood that it may be a founder mutation, although we are aware that further studies are necessary to verify this assumption.

The last, more frequent, BRCA1 PV in our cohort, c.4484G > T, was previously reported by other authors [31,33] in Brazil and in Portugal. What intrigues us regarding its presence in our region is that this PV is almost exclusive to the Taranto area and in particular to 2 specific towns of this province: Laterza and Ginosa. 

Among the more frequent BRCA2 PVs in Apulia, c.9676delT and c.8755-1G > A were enriched in the area of Lecce. As far as we know, only Santonocito et al. [25] reported these PVs in breast and/or ovarian cancer patients traveling to a Cancer Hospital in Rome which is a referral center for many Apulian patients, especially from Lecce. Therefore, it is reasonable to assume that the c.9676delT and the c.8755-1G > A mutations are of Apulian origin. We hope to support this hypothesis with future studies.

BRCA2 c.5796_5797delTA was mainly identified in several probands from areas of the Bari and BAT provinces, with significant enrichment in the BAT province. Interestingly, this variant seems to be very frequent in probands from the small town of Bisceglie. In the same town, the other PV encountered more frequently was the c.5266dupC in the BRCA1 gene.Instead, BRCA2:c.1796_1800delCTTAT is significantly enriched in the area of Bari and in particular in Bitetto, near Bari.

No peculiarities emerged in the genotype–phenotype correlation analysis. Breast and ovarian cancers were the most frequent malignancies described in our cohort. Of note was the fact that breast cancer cases were significantly more numerous in VUS-carriers than in PV-carriers and non-carriers. 

As previously reported, breast cancer is the most common malignancy in individuals with BRCA1 or BRCA2 germline PVs and the ductal histotype was more frequent than others. Lobular breast cancer was significantly more frequent in non-carriers than in carriers and VUS-carriers, thereby confirming that other genetic risk factors may underlie this type of cancer. As expected, among ovarian cancer probands, the number of PV-carriers was significantly higher than that of non-carriers and VUS-carriers, and the high grade serous histotype was the most frequent. An effective genotype–phenotype correlation analysis for Apulia calls for further studies that also include affected family members and not only probands.

## 5. Conclusions

We found that ten recurrent BRCA1/2 pathogenic variants account for more than half of the patients with proven HBOC syndrome from Apulia. Besides BRCA1 c.5266dupC, which is present in significant numbers in every Apulian province, the other PVs occur at a high frequency in some areas and not others. In-depth knowledge of the mutation spectrum of the target population and of the relatively small number of recurrent mutations is crucial to develop a specific cost-effective strategy for mutation screening and a program for breast–ovarian cancer control and prevention through more liberal, yet rational, genetic testing and counseling. Our findings should be explored in larger cohorts in order to assess whether recurrent mutation screening in Apulia could be effective. First, the cohort of the Foggia area, which was not included in our study due to problems with data collection, should be included in future studies. Second, a stepwide mutation screening protocol, based on initial screening for the Apulian common PVs, should be systematically applied in all breast/ovarian Apulian cancer patients and in all healthy Apulian people with a family history of breast/ovarian cancer, besides NCCN genetic test criteria. This information may represent a very small but necessary step for evaluating an Apulian population genetic screening (as Ashkenazi population) that can improve BRCA1/2 carriers’ identification deserving of a personalized preventive/therapeutic approach. Nowadays, our healthcare system is mainly focused on improving disease diagnosis and treatment instead of onillness prevention. In fact, current clinical practice for genetic testing is based on personal and/or familial cancer history: before targeting prevention for healthy family members, at least one of them must develop cancer. The development of new population-based genetic approaches can provide an impetus to increase carrier detection rates to maximize prevention and reduce cancer burden.

## Figures and Tables

**Table 1:** Probands-Description.

Feature	*n* (%)
SEX F M	1947 (96%) 79 (4%)
HEALTH STATE Healthy individuals at-risk Cancer patients	46 (2%) 1980 (98%)
AGE [mean (range)]	55 yrs. (21–92)
BRCA1/2 STATUS BRCA1/2 carriers BRCA1/2 non-carriers BRCA1/2 VUS	499 (24.6%) 1396 (69.1%) 131 (6.5%)
BRCA 1/2 carriers BRCA1 BRCA2	342 (68.4%) 158 (316%)

**Table 2:** Probands and BRCA1/2 status by Apulian province of birth.

Geographical Area of Origin	BRCA1/2 Status	Probands Tested *n* (%)
BARI	BRCA1carriers BRCA2 carriers BRCA1/2 non-carriers BRCA1/2 VUS TOTAL	105 (13%) 59(7%) 595 (72%) 63 (8%) 822
BAT (Barletta, Andria and Trani)	BRCA1carriers BRCA2 carriers BRCA1/2 non-carriers BRCA1/2 VUS TOTAL	53 (29%) 25 (14%) 101 (55%) 4 (2%) 182
BRINDISI	BRCA1carriers BRCA2 carriers BRCA1/2 non-carriers BRCA1/2 VUS TOTAL	25 (19%) 14 (10%) 90 (67%) 5 (4%) 134
FOGGIA	BRCA1carriers BRCA2 carriers BRCA1/2 non-carriers BRCA1/2 VUS TOTAL	10 (40%) 4 (16%) 6 (24%) 5 (20%) 25
LECCE	BRCA1carriers BRCA2 carriers BRCA1/2 non-carriers BRCA1/2 VUS TOTAL	103 (17%) 40 (6%) 422 (70%) 43 (7%) 618
TARANTO	BRCA1carriers BRCA2 carriers BRCA1/2 non-carriers BRCA1/2 VUS TOTAL	46 (19%) 16 (6%) 172 (70%) 11 (5%) 245

**Table 3:** Cancer type distribution relative to BRCA1/2 mutational status.

Cancer Type	Mutational Status	n (%)	*p*-Value
BC	C	358 (67.4)	
	NC	1102 (82.3)	**<0.0001**
	VUS	107 (84.9)	
Melanoma	C	0 (0)	
	NC	5 (0.37)	0.2
	VUS	1 (0.79)	
OC	C	144 (27.1)	
	NC	167 (12.4)	**<0.0001**
	VUS	17 (13.5)	
Pancreatic cancer	C	2 (0.4)	
	NC	5 (0.3)	0.7
	VUS	0 (0)	
Prostate cancer	C	1 (0,2)	
	NC	9 (0.7)	0.2
	VUS	0 (0)	
Other site	C	11 (2)	
	NC	21 (1.6)	0.2
	VUS	0 (0)	
Healthy	C	15 (2.8)	
	NC	30 (2.2)	0.3
	VUS	0 (0)	

**Table 4:** Breast cancer histological subtype and BRCA1/2 mutational status. C: carriers; NC: non-carriers; VUS: subjects carrying variant of uncertain significance.

Breast Cancer Histology	Mutational Status	n (%)	*p*-Value
**Ductal**	C	265 (84.4)	
	NC	774 (74.3)	**0.0009**
	VUS	70 (75.3)	
**in situ**	C	20 (6.4)	
	NC	102 (9.8)	0.1
	VUS	10 (10.7)	
**Lobular**	C	8 (2.5)	
	NC	80 (7.7)	**0.05**
	VUS	5 (5.3)	
**Mixed histology**	C	6 (1.9)	
	NC	26 (2.5)	0.6
	VUS	1 (1)	
**Other histologies**	C	15 (4.7)	
	NC	60 (5.8)	0.5
	VUS	7 (7.5)	

**Table 5:** Ovarian cancer histological subtype and BRCA1/2 mutational status.C: carriers; NC: non-carriers; VUS: subjects carrying variant of uncertain significance.

Ovarian Cancer Histology	Mutational Status	n (%)	*p*-Value
**Endometrioid**	C	15 (11.3)	
	NC	23 (14.1)	0.3
	VUS	4 (23.5)	
**High-grade serous**	C	109 (81.9)	
	NC	127 (77.9)	0.08
	VUS	10 (58.8)	
**Other histologies**	C	9 (6.7)	
	NC	13 (7.9)	**0.05**
	VUS	3 (17.6)	

**Table 6:** Recurrent PVs in BRCA1/2 carriers.

Gene	HGVS Nomenclature	Proband Numbers
BRCA1	c.5266dupC (p.Gln1756Profs)	188
BRCA1	c.65T > C (p.Leu22Ser)	19
BRCA1	c.514delC (p.Gln172fs)	14
BRCA1	c.1687C > T (p.Gln563Ter)	9
BRCA1	c.4484G > T (p.Arg1495Met)	7
BRCA2	c.5796_5797delTA (p.His1932Glnfs)	25
BRCA2	c.9676delT (p.Tyr3226Ilefs)	23
BRCA2	c.1796_1800delCTTAT (p.Ser599Terfs)	17
BRCA2	c.8755-1G > A	12
BRCA2	c.6462_6463delTC (p.Gln2157Ilefs)	10

**Table 7:** Frequencies of 10 recurrent PVs as reported in ExAC. European and global allele frequency and sample size of the ExAC cohort are reported. Regarding the BRCA2:c.1796_1800delCTTAT and BRCA2:c.8755-1G > A variants, the frequencies and sample size shown in the table refer to GnomAD Exomes (*) and the ALFA project (**), respectively. Allele frequencies in each Apulian province are reported and statistically compared. Population genetic frequencies has been retrieved from https://www.ncbi.nlm.nih.gov/snp/ on 20 October 2020. *p*-values refer to statistical comparison among Apulian provinces.

Recurrent PVs	European Allele Frequency	European Sample Size	Global Allele Frequency	Global Sample Size	Bari (%)	BAT (%)	Brindisi (%)	Foggia (%)	Lecce (%)	Taranto (%)	*p*-Value
BRCA1:c.1687C > T	0.00007	73,172	0.000041	121,058	6 (3)	1 (1)	0 (0)	0 (0)	2 (1)	0 (0)	n.s.
BRCA1:c.5266dupC	0.00026	73,354	0.000156	1,121,412	49 (29)	46 (59)	21 (53)	4 (28)	37 (25)	31 (50)	<0.0001
BRCA1:c.514delC	0.00001	73,352	0.000008	121,410	0 (0)	0 (0)	0 (0)	0 (0)	14 (9)	0 (0)	<0.0001
BRCA1:c.4484G > T	0.00001	73,324	0.000008	121,358	0 (0)	0 (0)	1 (2)	1 (8)	0 (0)	5 (8)	<0.0001
BRCA1:c.65T > C	/	/	/	/	12 (7)	0 (0)	0 (0)	0 (0)	7 (4)	0 (0)	0.018
BRCA2:c.5796_5797delTA	0.00001	73,246	0.000008	121,048	14 (8)	10 (12.9)	0 (0)	0 (0)	0 (0)	1 (1)	<0.0001
BRCA2:c.1796_1800delCTTAT	0.000008 *	132,644 *	0.000004 *	244,054 *	12 (7)	2 (2.5)	1 (2.5)	0 (0)	2 (1.4)	0 (0)	0.025
BRCA2: c.8755-1G > A	0.000008 **	16,160 **	0.00047 **	17,014 **	0 (0)	0 (0)	2 (5.1)	0 (0)	10 (7)	0 (0)	0.0009
BRCA2:c.6462_6463delTC	0.00001	72,338	0.000008	118,538	6 (3.6)	2 (2.6)	0 (0)	0 (0)	1 (0.7)	1 (1.6)	n.s.
BRCA2:C.9676delT	/	/	/	/	1 (0.6)	0 (0)	1 (2)	0 (0)	17 (11.8)	4 (6.4)	<0.0001

## Data Availability

Data are available upon reasonable request to corresponding author.

## Supplementary Materials

The following are available online at https://www.mdpi.com/article/10.3390/cancers13184714/s1, Table S1: list of identified VUS in probands; Table S2: Non coding PVs and large genomic rearrangements.
