# Supplementary material for: Spectrum of Germline Pathogenic Variants in BRCA1/2 Genes in the Apulian Southern Italy Population: Geographic Distribution and Evidence for Targeted Genetic Testing
Source: Cancers (Basel). 2021 Sep 21;13(18):4714. doi: 10.3390/cancers13184714 (PMC8467705; doi:10.3390/cancers13184714)
Supplement: Supplementary file 1 [file cancers-13-04714-s001.zip › cancers-1331888-supplementary.pdf]

cancers

MDPI

Article

Spectrum of Germline Pathogenic Variants in BRCA1/2 Genes in the Apulian Southern Italy Population: Geographic Distribution and Evidence for Targeted Genetic Testing

Margherita Patruno [1,*], Simona De Summa [2,*], Nicoletta Resta [3], Mariapia Caputo [2], Silvia Costanzo [1], Maria Digennaro [1], Brunella Pilato [2], Rosanna Bagnulo [3], Antonino Pantaleo [3], Cristiano Simone [3,4], Maria Iole Natalicchio [5], Elisabetta De Matteis [6], Paolo Tarantino [7], Stefania Tommasi [2] and Angelo Paradiso [1]

Supplementary materials:

Table S1. List of VUS identified in the analyzed cohort.

BRCA1	BRCA2
p.Pro1776Ser	p.Asp2566Glu
p.Arg1347Gly	p.Glu1811Ala
p.Arg1528Thr	p.Ile1298Thr
p.Asn1354Ser	p.Ala1170Val
p.Asp1152Asn	p.Ala1797Gly
p.Asp1435Gly	p.Ala1991Val
p.Asp67Tyr	p.Ala2351Thr
p.Asp96Gly	p.Ala3205Leu
p.Cys636Tyr	p.Arg155Thr
p.Gln1090Arg	p.Arg174His
p.Gln284Arg	p.Arg2108Cys
p.Gln284Arg	p.Arg2520Pro
p.Glu445Gln	p.Arg2787His
p.Glu761Gln	p.Arg3385His
p.Gly1201Cys	p.Asn1836Lys
p.His888Tyr	p.Asn1906ser
p.ILe1275Val	p.Asp125Glu
p.Lys970Asn	p.Asp191Asp
p.Pro1771Arg	p.Asp3112Asn
p.Pro568Leu	p.Asp3112Asn
p.Pro659Ala	p.Cys148Arg
p.Ser632Asn	p.Glu1110Gly
p.Ser784Leu	p.Glu1901Val
p.Thr333Ala	p.Glu2292Gly
p.Thr779Ala	p.Glu2961Ser
p.Val1378Ile	p.Glu2981Lys
p.Val1653Met	p.Glu3002asp
c.5468-10C>A	p.Gly2044Ala
c.67+8C>A	p.Gly267Glu
duplicazioneesone 3	p.His1966Arg
c.135-3T>C	p.Ile1237Met;
	p.Ile1244Thr
	p.Ile1298Met
	p.Ile982Leu

p.Leu914Phe
p.Lys2013Glu
p.Phe376Cys
p.Phe590Cys
p.Pro2589His
p.Pro2796Ser
p.Pro3194Gln
p.Ser1328Thr
p.Ser1468Thr
p.Ser1750Phe
p.Ser286Pro
p.Ser3366Asnfs*4
p.Thr1887Met
p.Thr200Ile
p.Thr2097Met
p.Thr2222Cys
p.Thr3288Ile
p.Thr64Ile
p.Tyr2624Cys
p.Val1810Ile
c.7435+6G>A
c.7805+6C>T
c.9502-12T>G
c.4211G>C
p.Ala1615Thr
p.Ala1615Thr
p.Arg278His

Table S2. Non coding PVs and Large Genomic Rearrangements (LGR).

BRCA1 non-coding PVs and LGRs	*n*
c.212+2T>A	2
c.547+2T>A	1
c.5153-1G>A	4
c.5278-2delA	2
exons 1-2 deletion	3
exons 1-12 deletion	2
exons 1-13 deletion	1
exons 1-20 deletion	1
promoter and exons 1-11 deletion	1
exon 7 deletion	1
exon 8 deletion	2
exon 19 deletion	1
exons 5-8 deletion	1
**BRCA2 non-coding PVs and LGRs**	***n***
c.67+1G>A	2
c.7976+1 G>A	1
c.8755-1G>A	12
exons 1-3 deletion	1
exon 14 deletion	1
